# Contact Allergy in the Elderly: A Study of 600 Patients

**DOI:** 10.3390/life12081228

**Published:** 2022-08-13

**Authors:** Dominik Németh, Györgyi Pónyai

**Affiliations:** 1 Department of Dermatology, Venereology and Dermato-Oncology, Semmelweis University, 41 Mária Street, 1085 Budapest, Hungary; 2 Károly Rácz Doctoral School of Clinical Medicine, Semmelweis University, 26 Üllői Street, 1085 Budapest, Hungary

**Keywords:** contact hypersensitivity, elderly, contact allergens, ageing

## Abstract

The proportion of elderly in the general population is increasing. Ageing of the skin and immune system can modify the features of contact hypersensitivity (CH). The number of epidemiological studies according to the age-related features of CH is very limited. We aimed to analyse the clinical characteristics of CH in an elderly patient population. A total of 600 patients (patient age > 60 years old) were patch tested with the European Environmental Baseline Series (EEBS) and 440 of them with the Complementary Fragrance Series (CFS) at the same time according to the actual international methodological standards in the Allergy Outpatient Unit of Department of Dermatology, Venereology and Dermato-Oncology of Semmelweis University between 2015–2019. Out of 600 tested patients, 54.8% had at least one allergen positivity. Female predominance was observed (78.7%). The most common diagnosis was contact dermatitis (63.7%), followed by psoriasis (6.2%). Most of the cases (58.0%) were found in the age group of 60–69. The five most common contact allergens were benzoic acid, methylisothiazolinone (MI), wood tar, nickel, and balsam of Peru. Allergic skin symptoms are present in all ages and also in the elderly. According to our data, the most common contact allergens are preservatives, followed by balsam of Peru among men and nickel among women. In case of contact dermatitis, stasis dermatitis, rosacea, and atopic dermatitis are worth patch testing to verify CH even in those above 60 years old.

## 1. Introduction

The proportion of the elderly especially in developed, welfare societies is gradually increasing. The “common image” of the elderly has changed recently. Unlike the older generation of previous decades, nowadays, they stay active longer and use cosmetics (anti-ageing products, make-ups, hair-dye) more often. At the same time, several skin diseases are also typically present, so not only long-term cosmetic but also therapeutic contact allergen exposure and thus the development of primary and secondary contact hypersensitivity (CH) is remarkable in this age group [1,2,3,4,5].

CH depends on several factors, including age. In the background of the increased susceptibility for CH, skin ageing plays a significant role. Intrinsic (e.g., genetic, metabolic, and hormonal processes) and extrinsic (UV radiation, mechanical damages, infections, toxic agents, pollutants) factors contribute to skin ageing. As a result of these factors, molecular and cellular changes occur with a clinical characteristic of dryer, inflexible, thinner skin and with slow and inadequate wound healing. Elderly skin becomes not only more prone to malignancies and infections, but allergen penetration is also easier through the damaged, impaired skin barrier. In spite of the decreasing age-related immunological reactions, the increased number and duration of environmental allergen exposures eventually make the development of CH possible [1,2,3,4]

## 2. Materials and Methods

In total, 1698 patients were patch tested consecutively in the Allergy Outpatient Unit of Department of Dermatology, Venereology and Dermato-Oncology of Semmelweis University in Hungary between 2015–2019 as the part of their examination for verifying CH based on their medical history. Out of those, patients who were at least 60 years old were involved in our study. Overall, 600 patients fulfilled this criterion. All of them were patch tested with the European Environmental Baseline Series (EEBS) and 440 patients with the Complementary Fragrance Series (CFS) at the same time as well according to their medical history. 

The used EEBS and CFS allergens were produced by Brial allergEAZE GmbH (Greven, Germany) (Table 1 and Table 2). The majority of the antigens was dissolved in Vaseline except formaldehyde, propylenglycol, Kathon CG^®^ (methylchloroisothiazolinone/methylisothiazolinone (MCI/MI) 3:1), and methylisothiasolinone (MI), which were used in the aqueous phase. The allergens were fixed on the patients’ asymptomatic back skin by Curatest plaster (Lohmann & Rauscher International GmbH & Co., KG D-56579 Rengsdorf, Germany).

The tests were made in accordance with international standards for patch tests in a 48-hour occlusion. Skin reactions were evaluated in 20–60 min, on day (D) D2, D3, D4, and on D7. Written informed consent was granted from all patients before performing patch tests. 

Data of patch tested patients were processed by two large groups: the total patient population of >60 years and the sensitised patient population of >60 years. These groups were distributed according to diagnosis, gender, and age groups (group of 60–69 years, group of 70–79 years, and group of >80 years). Among the sensitised patient population, the localisation of clinical symptoms and the most common contact allergens were also detected according to age groups.

## 3. Results

Out of the 1698 patch-tested patients between 2015–2019, the total patient population of >60 years consisted of 600 patients.

A total of 600 patients were patch tested with the EEBS, and 440 patients were also patch tested with CFS at the same time (Table 3).

### 3.1. Total >60-Year-Old Patient Population (n = 600)

- Overall, 54.8% (329 patients) of the entire patient population showed a positive reaction to at least one allergen (Table 4).

**Table 4:** Total >60-year-old patient population (*n* = 600) according to diagnosis and CH.

	Total >60 Years Patient Population (*n* = 600)				
*Diagnosis*	Total (Number)	Total (%)	Patients with CH (Number)	Patients with CH (%)	Patients with CH (%)
**Contact dermatitis**	382	63.7	237	72.0	62.0
**Atopic dermatitis**	11	1.8	9	2.7	81.8
**Psoriasis**	37	6.2	14	4.3	37.8
**Stasis dermatitis**	29	4.8	17	5.2	58.6
**Seborrheic dermatitis**	14	2.3	4	1.2	28.6
**Rosacea**	29	4.8	15	4.6	51.7
**Pruritus**	20	3.3	5	1.5	25.0
**Dyshidrosis**	18	3.0	6	1.8	33.3
**Microbial eczema**	17	2.8	7	2.1	41.2
**Urticaria**	7	1.2	3	0.9	42.9
**Other**	36	6.0	12	3.6	33.3
**Total**	600	100.0	329	100.0	54.8

Other: M. Hailey-Hailey, prurigo, lichen planopilaris, lichen planus erosiva genitalis, vasculitis, bullous pemphigoid. Sézary syndrome, lichen sclerosis et atrophicus, observatio, prurigo nodalaris, mycosis fungoides, lichen ruber planus, parapsoriasis, granuloma annulare, progressive systemic sclerosis, pemphigus vulgaris.

b. Proportion of diagnoses

Out of the 600 patients, 382 patients (63.7%) were diagnosed with contact dermatitis, which was the most common diagnosis, followed by psoriasis. (Table 4).

c. Gender distribution

According to the gender distribution, 472 patients were female (78.7%), 128 patients were male (21.3%), and the female:male ratio was about 3.5:1. (Table 5).

d. Age distribution

Among the 600 elderly patients, 348 patients (58.0%) belong to the age group of 60–69 years, 210 patients (35.0%) to 70–79 years, and 42 patients (7.0%) to >80 years (Table 6).

e. Proportion of diagnosis according to age groups

The most common diagnosis was contact dermatitis in every age group. In the age group 60–69, the second most common was psoriasis (6.6%); in the group of 70–79 years, the other diagnoses (7.6%); and in >80 years, the stasis dermatitis (14.3%) (Table 6).

### 3.2. Sensitised >60-Year-Old Patient Population (n = 329)

- Proportion of diagnoses

Among the 329 patients, the most common diagnosis was contact dermatitis in 237 patients (72.0%), followed by stasis dermatitis, rosacea, and psoriasis (Table 4).

b. Gender distribution

Female predominance was observed; female: 271 patients, male: 58 patients. In case of all genders, contact dermatitis was the most common diagnosis, followed by stasis dermatitis for men and rosacea for women (Table 5).

c. Distribution according to age groups and diagnosis

The majority of patients with CH (60.8%) belonged to the age group of 60–69 years, 32.2% to age group of 70–79 years, and 7.0% belonged to age group of >80 years. 

Contact dermatitis was the most common in all age groups, followed by stasis dermatitis and rosacea (age group 60–69), other diagnoses (age group of 70–79), and stasis dermatitis (age group >80 years) (Table 6).

d. Localisation of the clinical symptoms (Table 7)

In most cases, the upper limbs (50.5%) and the lower limbs (45.6%) were affected. Overall, 107 patients (32.5%) had symptoms on the face and 105 patients (31.9%) in the periorbital area. Furthermore, 26.4% of the patients had skin symptoms on the trunk. 

In the age group of 60–69 years, most skin symptoms appeared on the upper limb, lower limb, face, and periorbital region.

Among the age group of 70–79 years, the majority of skin symptoms were on the upper limbs and on the lower limbs, followed by trunk, facial, and periorbital localisations.

In case of the age group >80 years, the lower limb was the most common localisation, followed by the upper limb, the trunk, the anogenital-gluteal region, then the face, and the periorbital region.

### 3.3. Most Common Contact Allergens (Data of Table 3 were Used for Calculating)

- Sensitised >60-year-old patient population (*n* = 329)

The most common 20 allergens are shown in Table 8. Among these, the first was benzoic acid (16.1%), followed by MI, wood tar, nickel, and balsam of Peru. 

The benzoic acid was the most common contact allergen by men and women. Balsam of Peru was the second for male and MI for female patients.

b. Age group of 60–69 years with CH (*n* = 200)

The first 10 allergens were placed by 14 allergens (Table 9). The most common was benzoic acid (15.3%), followed by MI, nickel, wood tar, and balsam of Peru.

For men, MI and balsam of Peru were the most common allergens and for women the benzoic acid.

c. Age group of 70–79 years old (*n* = 106)

The most common allergen was benzoic acid (16.3%), followed by MI, wood tar, balsam of Peru, and nickel (Table 10).

For male patients, benzoic acid was the most common and for females the MI.

d. Age group of >80 years (*n* = 23)

The most common contact allergen was benzoic acid (22.6%), followed by wood tar, balsam of Peru, fragrance mix I, and fragrance mix II (Table 11). The most common contact allergen was benzoic acid for men and women as well. 

## 4. Discussion

Due to longer life expectancy, the number of elderly people shows an increase in the general population. The investigation of the age-specific characteristics of the skin and immune system has a several-decades-long history. However, the number of epidemiological studies regarding the connection of CH and old age is very limited. The studies of different working groups on CH do not reflect a common view on the concept of the onset of old age. The initial age and the examined age groups of their patient populations is different. The applied patch test series have also changed by the time, as new contact allergens were included in the series [1,2,3,4,5,6,7,8,9,10,11,12,13,14,15,16,17,18,19,20].

Allergic contact dermatitis (ACD) is a type-IV, cell-mediated CH reaction provoked by certain environmental contact allergens. ACD has two phases: the primer sensitisation to a certain allergen and the elicitation phase, which is the second contact with the allergen, when the clinical symptoms of the ACD appear [2]. 

CH depends on several factors, including age. Ageing affects every organ and cell in the body, including the skin and immune system as well. Certain intrinsic and extrinsic factors contribute to skin ageing. The former consists of different genetic, metabolic, and hormonal processes, while extrinsic factors such as UV radiation, mechanical damages, infections, toxic agents, cosmetics, foods, smoking, and air pollution can be mentioned [1,2]. 

Among intrinsic factors in genetics are crucial, progressive telomere shortening and the regulating effect of the microRNAs, which play a significant role. The natural moisturising factors (NMF), trans-epidermal water loss (TEWL), and the lipid content of the stratum corneum decreases with age, leading to dryer skin. For women, hormonal processes are also remarkable since postmenopausal women are more prone to skin drought. Regarding skin dryness, the number of sweat glands and sebaceous glands also drops. Structural changes of the skin also occurs: the skin becomes thinner, and the epidermal turnover time decreases. The extracellular matrix is also affected by ageing, with a reduced amount of glycosaminoglycans and hyaluronic acid content. Collagen synthesis is also disrupted because of the loss of fibroblasts, and degradation of elastin fibres is also remarkable. Disorganization and regression of small blood vessels and capillaries can be observed. Different kinds of environmental effects—most often in interaction—also play important roles regarding skin ageing. The previously mentioned extrinsic factors mainly cause changings in the DNA with the result of skin damage [1,3,4]. 

Regarding the ageing of the immune system, the term “immunosenescence” is used for defining every age-related change not only in the innate but also in the adaptive immunity of the human body. Although the number of receptors of the innate immune cells and the production of the proinflammatory cytokines is increased, the immune response is still not more intense. Dysfunction of monocytes/macrophages and neutrophils is present, impaired phagocytic capacity can occur. To summarise the age-dependent changes of the adaptive immunity, the decreased number and function of T cells should be highlighted. This change can be the consequence of the allergen-specific memory T-cell population increasing by ageing. Furthermore, the naive T-cell number decrease over the years, so there are gradually less T cells that can be stimulated by new allergens. Disrupted T-cell function is also caused by different age-dependent alterations of surface molecular expressions and internal signalling processes. Lymphopoiesis of B cells is also damaged: B-cell precursors and naive B cells are also affected by ageing. The antibodies produced by them have weaker affinity (because of the antibody isotype shift from IgG to IgM) and efficacy (elderly B cells can be stimulated less by dendritic cells) as well. Reduced production of IL-2 and the expression of CD40L of the elderly CD4+ T leads to disrupted interaction between B and T lymphocytes. Langerhans (LC) cells, which are the antigen-presenting cells in the skin, are fewer in number in the case of elderly people, which is supposedly the consequence of the reduced proliferation of the LC progenitors. Under normal circumstances, these cells migrate to the local lymph nodes to promote immune reactions and to activate the naive T-lymphocytes. By ageing, the mobilisation of LCs and the function to stimulate T cells is impaired. LCs also produce certain antimicrobial peptides in the epidermis, which regulates the tight junction formation in the human keratinocytes. Thus, the decreasing of the LCs by ageing also affects the normal epidermal skin barrier function [1,2,21,22,23,24,25].

Summarizing all the skin and immune system changes regarding age, it would be suspected that the decreased immune reactivity (Langerhans cell and T-lymphocyte number and function) would block the development of CH. However, CH is still possible and worth discussing because of the impaired skin barrier and the longer and more diverse environmental contact allergen exposure. CH can be a primer-provoking factor (allergic contact dermatitis) or may connect to other diagnoses (e.g., atopic dermatitis, stasis dermatitis, rosacea, psoriasis, seborrheic dermatitis, dyshidrosis, pruritus) as a secondary exacerbating factor [1,2,3,4,26,27,28].

To our best knowledge, our study is the most recent European investigation to examine the characteristics of CH in the elderly (after the official inclusion of MI to the EEBS, and with the Hungarian CFS allergens patch tested). The aim of our study was to analyse the data of the total patient population of >60 years and the sensitised patient population of >60 years according to age, gender, diagnosis, CH, and skin symptom localisations.

According to our data, out of the total patch-tested >60-year-old patient population (*n* = 600), 329 patients (54.8%) had CH. Among them, the most common diagnosis was contact dermatitis, followed by stasis dermatitis. Atopic dermatitis, pruritus, dyshidrosis, microbial eczema, and urticaria occurred less commonly. *Mahler* found that allergic contact dermatitis was also the most common disease among patients above 65 years old within the entire patient population. The third diagnosis was stasis dermatitis/ulcus cruris, which was also a common disease in our own study. At the 6th place, in accordance with our data, atopic dermatitis was found [29].

In our examination, CH was found in a higher rate in contact dermatitis, stasis dermatitis, rosacea, and atopic dermatitis. This observation shows it is worth patch testing elderly patients with these four diagnoses. 

Epidemiological studies of CH in the elderly published before 2010 reported fragrances (fragrance mix, balsam of Peru), metals (nickel, chromium, cobalt, mercury), topical antibacterial compounds (neomycin), rubber accelerators, and paraphenylenediamine as the most common contact allergens [2,5,10,12,13,14,15,27,30]. In 2011, *Balato* et al. highlighted the importance of lanolin alcohols, paraben mix, diamino diphenylmethane, quinolone mix, and methyldibromo-glutaronitrile (Euxyl K400) [2]. In 2015, *Mahler* found fragrance mix I the most common contact allergen by the over-65-year-old patient group, followed by balsam of Peru, nickel, fragrance mix II, colophonium, propolis, Kathon CG^®^, lanolin alcohol, amerchol 101 and tert, butylhydrochinon. MI was only the 16th allergen among the above-65-year-old patients [29]. A recent North American study found balsam of Peru as the most common contact allergen in the elderly (aged >65 years), followed by MI, nickel, hydroperoxide of linalool, and fragrance mix I. [28].

Mentioning the age-related trends of contact dermatitis, the nickel frequency in the elderly is usually interpreted by sensitisation from younger age, according to the literature, and is commonly referred to as having a decreasing tendency. The prevalence of sensitisation frequencies to topical medications is described with an increasing tendency in ageing mainly because of the longer duration of exposure. Fragrance mix, balsam of Peru, neomycin, gentamicin, and lanolin are mentioned by the literature. Topical corticosteroid contact allergy should also be taken into account at older ages [2,6]. 

Regarding our own data, in the patient population of >60 years (*n* = 600), we found benzoic acid as the most common contact allergen (16.1%), followed by MI (13.2%), wood tar (10.8%), and nickel (10.7%). Compared to the literature, in our study, the most common ones were the preservatives (benzoic acid part of CFS and MI, tested from 2014) and not the fragrances or metals. The high sensitisation frequency of benzoic acid can be a consequence of the typical Hungarian cuisine and preservation of traditions since it is commonly used in different kinds of compotes and conserves that can not only be purchased at shops but can also be produced by people at home. 

According to allergen frequencies in all age-groups *benzoic acid* was the most common. *MI* was the second in age groups 60–69 and 70–79. *Wood tar* was observed with gradually increasing rates by ageing. Regarding *nickel*, a decreasing tendency was found in ageing: only one patient had nickel CH in the group >80 years. This decreasing characteristic correlates with the data of the literature [5,13,15].

In case of fragrances, *balsam of Peru* became more frequent from the age group 60–69 to the age group >80 years. *Fragrance mix* *I* and *fragrance mix II* had higher frequencies from the age group 70–79 to >80 years. This increasing frequency of fragrance CH is also reported in the literature [2,31]. Rates of *lanolin alcohol, propylenglycol,* and *neomycin,* which are common components of several local therapeutics, are also remarkable, highlighting their use in all age groups. 

According to our data among the sensitised patient population of >60 years (*n* = 329), most skin symptoms appeared on the limbs, face/periorbital region, and trunk. *Onder* et al. also found common arm, hand, leg, face, neck, and trunk involvement in the elderly [30]. *Balato* et al. reported widespread skin symptoms and hand and lower limb involvement among elderly patients with CH [5]. In the over-65-year-old overall patient population, *Mahler* found leg involvement and often widespread facial and oral mucosal symptoms [29]. According to *Goh* et al., contact dermatitis usually affects the lower extremities of the elderly patients, while face and hand involvement is typical among younger patients. It was explained by the localisation of medical conditions of the elderly (e.g., stasis dermatitis) and occupational and cosmetic-related sources of exposures in the case of younger individuals [6,20]. In contrast to this, our results showed common affection of upper limb, face, and periorbital regions. This finding can be a result of the fact that the elderly people of our time stay more active and use cosmetics, anti-ageing products, and also keep their occupation longer. 

Our five-year retrospective study of 600 (>60 years) patients aimed to observe the clinical features of CH in the elderly.

More than half of the total elderly (>60 years) patient population (*n* = 600) had at least one CH. Most cases were detected in the age group of 60–69 years. The most common diagnosis was contact dermatitis, followed by psoriasis. According to our data, in the case of contact dermatitis, stasis dermatitis, atopic dermatitis, and rosacea, patch testing is worth performing. Female predominance was observed in every age group. For men, after benzoic acid, balsam of Peru is the second most common contact allergen, and for women, preservatives were followed by nickel. For sensitised male patients stasis dermatitis and for sensitised female patients rosacea followed the contact dermatitis. Most symptoms appeared on limbs and face/periorbital region. We detected similar distribution of clinical symptom localisation among the 60–69-year-old and 70–79-year-old patients. Preservatives were the most common contact allergens in every age group. MI CH was less frequent in the age group of >80 years. It can be the result of MI appearing as a new allergen around the 2000s, so these patients spent most of their lives without this allergen exposure. In our study, fragrance CH increased and nickel CH decreased with age, which correlates with international investigations.

In conclusion, CH in the elderly can be carried over from younger age, but new CH can develop as well from workplaces, leisure and home activities, daily cosmetics, household chemicals, and external topicals used to treat skin diseases, systemic treatments, previous surgical history (e.g., orthopaedic/dental implants, prostheses). CH must be taken into account in old age, and patch testing is recommended for diagnosis even above 80 years of age.

Limitation of the study: This is a cross-sectional picture of the current status of the patients’ CHs when the patch test was performed. The exact timepoint when the patients developed the CH cannot be established accurately. It may be that the CH was carried over from younger ages or developed as a new CH during older age.

## Figures and Tables

**Table 1:** European Environmental Baseline Series (EEBS) allergens (2015–2019).

	Allergen (Concentration)	Period
1.	Neomycin-sulphate (20%)	2015-
2.	Benzocaine (5%)	2015-
3.	Jodchlore-oxychinoline (clinoquinol) (5%)	2015-
4.	Paraben mix (16%)	2015-
5.	Lanoline alcohol (30%)	2015-
6.	Primin (0.01%)	2015-
7.	Sesquiterpen lactone (0.1 %)	2015-
8.	Phenylbutazone (10%)	2015-
9.	Potassium dichromate (0.5%)	2015-
10.	Nickel (II)-sulphate hexahydrate (5%)	2015-
11.	Cobalt (II)-chloride hexahydrate (1%)	2015-
12.	Thiuram mix (1%)	2015-
13.	2-Mercaptobenzothiazole (MBT) (2%)	2015-
14.	Colophonium (20%)	2015-
15.	Wood tar (12%)	2015-
16.	Balsam of Peru (*Myroxylone pereirae*) (25%)	2015-
17.	Lyral^®^ (Hidroxyisohexyl 3-cyclohexene carboxaldehyde) (5%)	2018-
18.	PPD (4-phenylendiamine base) (1%)	2015-
19.	Mercury (II)-amidochloride (1%)	2015-
20.	Formaldehyde (2%)	2015-
21.	*n*-isopropyl-N’-phenyl-*p*-phenylendiamine (IPPD) (0.1%)	2015-
22.	Propylenglycol (20%)	2015-
23.	Thiomersal (0.1%)	2015-
24.	Quaternium 15 (Dowicil 200) (1%)	2015-
25.	Kathon CG^®^ (methylchloroisothiazolinon/methylisothiazolinone (MCI/MI) 3:1) (0.01%)	2015-
26.	Resorcin (2%)	2015-
27.	Propolis (10%)	2015-
28.	*p*-tert-butylphenol-formaldehyde-resin (1%)	2015-
29.	Fragrance mix I (8%)	2015-
30.	Mercury-chloride (0.1%)	2015-
31.	Bisphenol A (epoxy resin) (1%)	2015-
32.	Budesonide (0.1%)	2015-
33.	Tixocortol-21-pivalate (1%)	2015-
34.	Methyldibromo-glutaronitrile (MDBGN) (0.3%)	2015-
35.	Fragrance mix II (14%)	2015-
36.	Lavender oil (2%)	2015-
37.	Methylisothiazolinone (MI) (0.2%)	2015-
38.	2-hydroxyethyl-methacrylate (2%)	2017- (98 patients in 2016)
39.	Methyl-methacrylate (2%)	2017- (98 patients in 2016)
40.	Ethyl-acrylate (0.1%)	2017- (98 patients in 2016)
41.	Cocamidopropyl betaine (1%)	2015-
42.	d-Limonene (10%)	24/10/2017-
43.	Linalool (10%)	24/10/2017-
44.	Lauryl-glycoside (3%)	2018-
45.	Decyl-glycoside (5%)	2018-
46.	Sorbitan sesquioleate (20%)	16/08/2019-
47.	Turpentine oil (0.3%)	2015–2017
48.	*Evernia furfuracea* (tree moss) (1%)	19/02/2018-

**Table 2:** Complementary Fragrance Series (CFS) allergens (2015–2019).

	Allergen (Concentration)	Period
1.	Benzoic acid (5%)	2015-
2.	Cinnamon oil (0.5%)	2015-
3.	Vanilla (10%)	2015-
4.	Camphor (1%)	2015-
5.	Menthol (1%)	2015-
6.	Sorbic acid (2%)	2015-

**Table 3:** Distribution of patients patch tested with EEBS (*n* = 600) and CFS (*n* = 440) according to gender and age (number of patients).

	EEBS			CFS		
	Total	Male	Female	Total	Male	Female
**60–69 years**	348	72	276	262	50	212
**70–79 years**	210	46	164	147	33	114
**>80 years**	42	10	32	31	6	25
**Total**	600	128	472	440	89	351

**Table 5:** Total (*n* = 600) and sensitised (*n* = 329) >60-year-old patient population according to diagnosis and gender.

	Total >60-Year-Old Patient Population (*n* = 600)				Sensitised >60-Year-Old Patient Population (*n* = 329)			
*Diagnosis*	Male (Number)	Male (%)	Female (Number)	Female (%)	Male (Number)	Male (%)	Female (Number)	Female (%)
**Contact dermatitis**	67	52.3	315	66.7	37	63.8	200	73.8
**Atopic dermatitis**	5	3.9	6	1.3	4	6.9	5	1.8
**Psoriasis**	9	7.0	28	5.9	5	8.6	9	3.3
**Stasis dermatitis**	12	9.4	17	3.6	6	10.3	11	4.1
**Seborrheic dermatitis**	3	2.3	11	2.3	0	0.0	4	1.5
**Rosacea**	3	2.3	26	5.5	1	1.7	14	5.2
**Pruritus**	7	5.5	13	2.8	0	0.0	5	1.8
**Dyshidrosis**	2	1.6	16	3.4	0	0.0	6	2.2
**Microbial eczema**	6	4.7	11	2.3	2	3.4	5	1.8
**Urticaria**	1	0.8	6	1.3	1	1.7	2	0.7
**Other**	13	10.2	23	4.9	2	3.4	10	3.7
**Total**	128	100.0	472	100.0	58	100.0	271	100.0

Other: M. Hailey-Hailey, prurigo, lichen planopilaris, lichen planus erosiva genitalis, vasculitis, bullous pemphigoid, Sézary syndrome, lichen sclerosus et atrophicus, observatio, prurigo nodalaris, mycosis fungoides, lichen ruber planus, parapsoriasis, granuloma annulare, progressive systemic sclerosis, pemphigus vulgaris.

**Table 6:** Total (*n* = 600) and sensitised (*n* = 329) >60-year-old patient population according to diagnosis and age groups.

	Total >60-Year-Old Patient Population (*n* = 600)						Sensitised >60-Year-Old Patient Population (*n* = 329)					
	60–69 Years		70–79 Years		>80 Years		60–69 Years		70–79 Years		>80 Years	
*Diagnosis*	Number	%	Number	%	Number	%	Number	%	Number	%	Number	%
**Contact dermatitis**	227	65.2	132	62.9	23	54.8	144	72.0	80	75.5	13	56.5
**Atopic dermatitis**	6	1.7	4	1.9	1	2.4	5	2.5	3	2.8	1	4.3
**Psoriasis**	23	6.6	10	4.8	4	9.5	9	4.5	3	2.8	2	8.7
**Stasis dermatitis**	14	4.0	9	4.3	6	14.3	10	5.0	3	2.8	4	17.4
**Seborrheic dermatitis**	9	2.6	4	1.9	1	2.4	3	1.5	0	0.0	1	4.3
**Rosacea**	18	5.2	11	5.2	0	0.0	10	5.0	5	4.7	0	0.0
**Pruritus**	7	2.0	11	5.2	2	4.8	3	1.5	2	1.9	0	0.0
**Dyshidrosis**	15	4.3	2	1.0	1	2.4	5	2.5	1	0.9	0	0.0
**Microbial eczema**	6	1.7	9	4.3	2	4.8	4	2.0	3	2.8	0	0.0
**Urticaria**	5	1.4	2	1.0	0	0.0	3	1.5	0	0.0	0	0.0
**Other**	18	5.2	16	7.6	2	4.8	4	2.0	6	5.7	2	8.7
**Total**	348	100.0	210	100.0	42	100.0	200	100.0	106	100.0	23	100.0

Other: M. Hailey-Hailey, prurigo, lichen planopilaris, lichen planus erosiva genitalis, vasculitis, bullous pemphigoid, Sézary syndrome, lichen sclerosus et atrophicus, observatio, prurigo nodalaris, mycosis fungoides, lichen ruber planus, parapsoriasis, granuloma annulare, progressive systemic sclerosis, pemphigus vulgaris.

**Table 7:** Sensitised >60-year-old patient population (*n* = 329) according to clinical symptom localisation.

	Sensitised >60-Year-Old Patient Population (*n* = 329)			
*Clinical Localisation (%)*	Total (*n* = 329)	60–69 Years (*n* = 200)	70–79 Years (*n* = 106)	>80 Years (*n* = 23)
**Face**	32.5	35.0	29.2	26.1
**Periorbital region**	31.9	34.5	28.3	26.1
**Scalp**	18.5	19.5	16.0	21.7
**Neck**	21.0	21.5	20.8	17.4
**Upper limbs**	50.5	50.0	52.8	43.5
**Lower limbs**	45.6	41.0	51.9	56.5
**Trunk**	26.4	24.0	30.2	30.4
**Anogenital-gluteal region**	19.8	17.5	21.7	30.4
**Other (mouth, ears, bends)**	23.1	23.0	23.6	21.7
**Generalised**	14.9	15.0	14.2	17.4

**Table 8:** Most common contact allergens of the sensitised >60-year-old patient population (*n* = 329) by gender.

		Sensitised >60-Year-Old Patient Population (*n* = 329)					
		Total (*n* = 329)		Male (*n* = 58)		Female (*n* = 271)	
	Allergen	Number	%	Number	%	Number	%
**1.**	**Benzoic acid**	71	16.1	16	18.0	55	15.7
**2.**	**Methylisothiazolinone**	79	13.2	16	12.5	63	13.3
**3.**	**Wood tar**	65	10.8	10	7.8	55	11.7
**4.**	**Nickel**	64	10.7	4	3.1	60	12.7
**5.**	**Balsam of Peru**	58	9.7	18	14.1	40	8.5
**6.**	**Fragrance mix I**	49	8.2	12	9.4	37	7.8
**7.**	**Propylenglycol**	40	6.7	12	9.4	28	5.9
**8.**	**Fragrance mix II**	39	6.5	6	4.7	33	7.0
**9.**	**Kathon CG** **^®^**	34	5.7	5	3.9	29	6.1
**10.**	**Lanolin alcohol**	33	5.5	11	8.6	22	4.7
**11.**	**Sorbic acid**	22	5.0	4	4.5	18	5.1
**12.**	**Propolis**	25	4.2	4	3.1	21	4.4
	**Potassium dichromate**	25	4.2	2	1.6	23	4.9
	**Neomycin-sulphate**	25	4.2	4	3.1	21	4.4
**13.**	**Mercury (II)-amidochloride**	18	3.0	1	0.8	17	3.6
**14.**	**Cobalt chloride**	17	2.8	1	0.8	16	3.4
**15.**	**PPD**	16	2.7	2	1.6	14	3.0
**16.**	**Budesonide**	14	2.3	1	0.8	13	2.8
**17.**	**Formaldehyde**	12	2.0	3	2.3	9	1.9
	**Colophonium**	12	2.0	3	2.3	9	1.9
**18.**	**Methyldibromo-glutaronitrile**	11	1.8	5	3.9	6	1.3
	**Cocamidopropyl-betaine**	11	1.8	5	3.9	6	1.3
	**Mercury-chloride**	11	1.8	0	0.0	11	2.3
**19.**	**Paraben mix**	10	1.7	4	3.1	6	1.3
**20.**	**Thiomersal**	9	1.5	1	0.8	8	1.7
	**Thiuram mix**	9	1.5	1	0.8	8	1.7

Data of Table 3 were used for calculating: for complementary fragrance series allergens, the total population consisted of 440 patients.

**Table 9:** Most common contact allergens of the sensitised 60–69-year-old patient population (*n* = 200) by gender.

		Sensitised 60–69-Year-Old Patients (*n* = 200)					
		Total (*n* = 200)		Male (*n* = 35)		Female (*n* = 165)	
	Allergen	Number	%	Number	%	Number	%
**1.**	**Benzoic acid**	40	15.3	6	12.0	34	16.0
**2.**	**Methylisothiazolinone**	47	13.5	10	13.9	37	13.4
**3.**	**Nickel**	46	13.2	2	2.8	44	15.9
**4.**	**Wood tar**	38	10.9	4	5.6	34	12.3
**5.**	**Balsam of Peru**	34	9.8	10	13.9	24	8.7
**6.**	**Fragrance mix I**	29	8.3	5	6.9	24	8.7
**7.**	**Fragrance mix II**	21	6.0	3	4.2	18	6.5
	**Propylenglycol**	21	6.0	7	9.7	14	5.1
**8.**	**Kathon CG** **^®^**	20	5.7	4	5.6	16	5.8
	**Lanolin alcohol**	20	5.7	8	11.1	12	4.3
**9.**	**Potassium dichromate**	16	4.6	2	2.8	14	5.1
	**Sorbic acid**	12	4.6	2	4.0	10	4.7
**10.**	**Propolis**	14	4.0	1	1.4	13	4.7
	**Neomycin-sulphate**	14	4.0	3	4.2	11	4.0

Data of Table 3 were used for calculating: for complementary fragrance series allergens, the total population consisted of 440 patients.

**Table 10:** Most common contact allergens of the sensitised 70–79-year-old patient population (*n* = 106) by gender.

		Sensitised 70–79-Year-Old Patients (*n* = 200)					
		Total (*n* = 106)		Male (*n* = 20)		Female (*n* = 86)	
	Allergen	Number	%	Number	%	Number	%
**1.**	**Benzoic acid**	24	16.3	9	27.3	15	13.2
**2.**	**Methylisothiazolinone**	30	14.3	6	13.0	24	14.6
**3.**	**Wood tar**	20	9.5	5	10.9	15	9.1
**4.**	**Balsam of Peru**	18	8.6	7	15.2	11	6.7
**5.**	**Nickel**	17	8.1	2	4.3	15	9.1
**6.**	**Propylenglycol**	16	7.6	5	10.9	11	6.7
**7.**	**Fragrance mix I**	15	7.1	7	15.2	8	4.9
**8.**	**Fragrance mix II**	14	6.7	2	4.3	12	7.3
**9.**	**Kathon CG** **^®^**	13	6.2	1	2.2	12	7.3
**10.**	**Sorbic acid**	9	6.1	2	6.1	7	6.1

Data of Table 3 were used for calculating: for complementary fragrance series allergens, the total population consisted of 440 patients.

**Table 11:** Most common contact allergens of the sensitised >80-year-old patient population (*n* = 23) by gender.

		Sensitised >80-Year-Old Patients (*n* = 23)					
		Total (*n* = 23)		Male (*n* = 3)		Female (*n* = 20)	
	Allergen	Number	%	Number	%	Number	%
**1.**	**Benzoic acid**	7	22.6	1	16.7	6	24.0
**2.**	**Wood tar**	7	16.7	1	10.0	6	18.8
**3.**	**Balsam of Peru**	6	14.3	1	10.0	5	15.6
**4.**	**Fragrance mix I**	5	11.9	0	0.0	5	15.6
**5.**	**Fragrance mix II**	4	9.5	1	10.0	3	9.4
**6.**	**Lanolin alcohol**	3	7.1	1	10.0	2	6.3
	**Propylenglycol**	3	7.1	0	0.0	3	9.4
**7.**	**Methylisothiazolinone**	2	4.8	0	0.0	2	6.3
	**Colophonium**	2	4.8	1	10.0	1	3.1
	**Propolis**	2	4.8	0	0.0	2	6.3
	**Benzocaine**	2	4.8	0	0.0	2	6.3
**8.**	**Sorbic acid**	1	3.2	0	0.0	1	4.0
	**Vanilla**	1	3.2	0	0.0	1	4.0
	**Menthol**	1	3.2	0	0.0	1	4.0
**9.**	**Paraben mix**	1	2.4	0	0.0	1	3.1
	**Kathon CG** **^®^**	1	2.4	0	0.0	1	3.1
	**PPD**	1	2.4	0	0.0	1	3.1
	**IPPD**	1	2.4	0	0.0	1	3.1
	**Resorcin**	1	2.4	0	0.0	1	3.1
	**Mercury-chloride**	1	2.4	0	0.0	1	3.1
	**Mercury(II)-amidochloride**	1	2.4	1	10.0	0	0.0
	**Nickel**	1	2.4	0	0.0	1	3.1
	**Epoxy resin**	1	2.4	0	0.0	1	3.1
	**Budesonide**	1	2.4	0	0.0	1	3.1
	**Neomycin-sulphate**	1	2.4	0	0.0	1	3.1

Data of Table 3 were used for calculating: for complementary fragrance series allergens, the total population consisted of 440 patients.
